# Regulatory Networks of Non-Coding RNAs Modulating Natural Killer Cell Antitumor Immunity in the Tumor Microenvironment

**DOI:** 10.3390/cells15141260

**Published:** 2026-07-13

**Authors:** Zida Xu, Can Jin, Xuan Huang

**Affiliations:** 1 The MOE Basic Research and Innovation Center for the Targeted Therapeutics of Solid Tumors, The Queen Mary School, Jiangxi Medical College, Nanchang University, Nanchang 330031, China; 8101125116@email.ncu.edu.cn; 2 Jiangxi Provincial Key Laboratory of Bioengineering Drugs, Institute of Translational Medicine, Jiangxi Medical College, Nanchang University, Nanchang 330031, China; 407400250003@email.ncu.edu.cn; 3 Chongqing Research Institute, Nanchang University, Chongqing 400010, China

**Keywords:** natural killer (NK) cells, tumor microenvironment (TME), non-coding RNAs (ncRNAs), competitive endogenous RNA (ceRNA), regulatory mechanism, cancer immunotherapy

## Abstract

The intricate intercellular communication within the tumor microenvironment (TME) critically drives cancer progression and therapeutic resistance. Natural killer (NK) cells are potent sentinels of the innate immune system, but their antitumor functions are often severely compromised by the TME’s immunosuppressive networks. Moving beyond protein-coding genes, non-coding RNAs (ncRNAs)—with microRNAs (miRNAs) playing a foundational role alongside long non-coding RNAs (lncRNAs) and circular RNAs (circRNAs)—have emerged as vital components of the regulatory networks influencing immune responses. Rather than dictating immune cell fate, these diverse transcriptomic classes form complex networks that modulate NK cell functional states and TME immunosuppression. This review systematically elucidates the molecular mechanisms by which these ncRNA networks influence NK cell biology in the TME. We dissect three core regulatory axes driven by extracellular vesicle (EV)-mediated communication, competitive endogenous RNA crosstalk, and epigenetic remodeling: the extrinsic suppression of NK cells by EV-derived and secreted ncRNAs from TME-resident cells, the reciprocal modulation of TME components by NK cell-derived ncRNAs, and the intrinsic regulation of NK cell functions by endogenous ncRNAs. Furthermore, we critically assess the clinical translational potential of targeting these networks. We highlight specific ncRNAs as non-invasive prognostic biomarkers and summarize targeted therapeutic interventions using antisense oligonucleotides, small interfering RNAs, and nano-delivery systems. Modulating these core ncRNA nodes to mitigate TME immunosuppression offers a novel paradigm for precision oncology, holding substantial promise for enhancing immune checkpoint blockade and NK cell-directed immunotherapies.

## 1. Introduction

Cancer remains a leading cause of global mortality, with increasing incidence driven by aging populations and environmental factors. While targeted therapy and immunotherapy have revolutionized treatment [1], intrinsic/extrinsic resistance and tumor heterogeneity still limit clinical outcomes [2]. Accumulating evidence highlights the tumor microenvironment (TME) as a key determinant of cancer progression, making the dissection of intercellular regulatory networks critical for developing novel therapeutic strategies.

The TME is no longer perceived as a passive bystander in carcinogenesis but is now recognized as a dynamic and intricate ecosystem that is pivotal to tumor progression, metastasis, and therapeutic resistance [3]. This complex milieu is composed of tumor cells, a diverse array of immune cells, and stromal components, such as cancer-associated fibroblasts (CAFs) and endothelial cells [4], all engaged in a continuous and reciprocal cellular dialogue. Biologically, the TME functions as a highly cooperating network that actively dictates tumor trajectory through several core functions [5]. First, it drives tumor angiogenesis by secreting pro-angiogenic factors, establishing a vascular network that supplies oxygen and nutrients to proliferating cancer cells. Second, the TME facilitates extracellular matrix (ECM) remodeling, wherein enzymes like matrix metalloproteinases modify tissue stiffness and architecture to clear physical paths for tumor invasion and metastasis [6]. Third, it induces profound metabolic reprogramming; the TME is characteristically hypoxic, acidic, and nutrient-depleted, a condition that metabolically adapted tumor cells exploit for survival while simultaneously starving infiltrating immune cells [7]. Most importantly, the TME exerts robust immunosuppressive functions. Through the secretion of suppressive cytokines (such as TGF-β and IL-10) and the recruitment of regulatory populations like myeloid-derived suppressor cells (MDSCs) and regulatory T cells (Tregs), the TME actively paralyzes the cytotoxic capabilities of immune effectors, establishing a protective niche for immune evasion [5,7].

Within this network, Natural Killer (NK) cells function as critical sentinels of the innate immune system [8]. Their capacity to recognize and eliminate transformed cells without prior sensitization positions them as powerful effectors of cancer immunosurveillance. NK cell function is regulated by a sophisticated balance of activating and inhibitory receptors that detect “altered self” or “missing self” signatures on target cells [9]. Upon activation, they execute potent cytotoxic responses through the release of perforin and granzymes, engagement of death receptors, and secretion of pro-inflammatory cytokines such as IFN-γ, which further modulates broader antitumor immunity [10].

Beyond direct cytotoxicity, NK cells exert multifaceted immunomodulatory functions. As a primary source of IFN-γ, they enhance antigen presentation, promote Th1 polarization, and inhibit tumor angiogenesis [11]. However, their functional plasticity allows them to adopt diverse phenotypic states in response to environmental cues. Notably, the formidable antitumor potential of NK cells is frequently subdued within the TME. Tumors employ diverse evasion strategies, including the shedding of activating ligands, the upregulation of inhibitory molecules, and the recruitment of immunosuppressive cells. Furthermore, metabolic constraints such as nutrient competition and accumulation of immunosuppressive metabolites further compromise NK cell recruitment and effector fitness [11,12]. This paradoxical coexistence of potent effector mechanisms and functional inhibition underscores the necessity to decipher the molecular dialogue regulating NK cell activity in the TME.

For decades, the regulatory landscape of the TME was largely viewed through the lens of protein-coding genes. This perspective has been fundamentally reshaped by the discovery of non-coding RNAs (ncRNAs) [13]. Once dismissed as transcriptional “noise,” ncRNAs are now acknowledged as crucial components in gene regulatory networks. The repertoire of ncRNAs includes well-characterized microRNAs (miRNAs), which post-transcriptionally silence target mRNAs, as well as long non-coding RNAs (lncRNAs) and circular RNAs (circRNAs) [14], which exhibit astonishing structural and functional diversity. These endogenous and extracellular ncRNAs can modulate gene expression through a myriad of mechanisms, including chromatin remodeling, transcriptional interference, and by participating in competitive endogenous RNA (ceRNA) networks—a complex web of interactions where transcripts like lncRNAs and circRNAs act as molecular sponges for miRNAs, thereby de-repressing mRNA targets [15]. While the roles of miRNAs in the TME are increasingly mapped, the broader universe of lncRNAs and circRNAs and their specific roles in sculpting the stromal-immune interface, particularly concerning NK cell biology, remain a vast and largely uncharted territory.

This review aims to bridge this critical knowledge gap by moving beyond the conventional focus on miRNAs and unidirectional regulation. We will systematically decode the sophisticated mechanisms by which a wider spectrum of ncRNAs—primarily lncRNAs and circRNAs—modulate three core regulatory axes in the TME: (1) extracellular vesicle (EV)-derived and secreted ncRNAs from TME-resident cells suppressing NK cell function; (2) NK cell-derived ncRNAs modulating reciprocal TME components; and (3) endogenous ncRNAs governing NK cell intrinsic biology. Our discourse will dissect how these ncRNAs, shuttled via extracellular vesicles or acting intracellularly, modulate key signaling pathways, cytokine networks, and metabolic programs. Furthermore, we will synthesize the intricate regulatory networks woven by these ncRNAs, highlighting the pivotal role of ceRNA circuits and epigenetic regulation. Finally, we will critically assess the translational potential of targeting these ncRNAs across all three axes to reprogram the TME from an immunosuppressive to an immunostimulatory state, discussing their promise as novel biomarkers and therapeutic targets to bolster the efficacy of cancer immunotherapy, with a specific vision for enhancing NK cell-based treatment modalities. To maintain a rigorous and focused discourse, it is imperative to delineate the specific scope and inherent limitations of this review. Although we emphasize the regulatory networks governing pivotal pathways and specific receptors, such as NKG2D and DNAM1, the modulation of other activating receptors, including NKp30, NKp44, and NKp46, extends beyond our current purview. Additionally, this discussion does not encompass the regulatory mechanisms underlying classical inhibitory HLA receptors involving NKG2A/CD94 and CLIR and their corresponding activating isoforms. Furthermore, while the TME houses a diverse innate immune repertoire, our analysis remains strictly confined to NK cells and does not address the influence of non-coding RNAs on other innate lymphoid cell (ILC) populations. By establishing these precise boundaries and providing a highly focused, mechanistic overview, this review seeks to establish a new paradigm for understanding immune evasion and to illuminate novel therapeutic avenues in oncology.

## 2. Core Molecular Mechanisms of ncRNA-Mediated Regulation

The sophisticated dialogue within the tumor microenvironment is regulated by a complex molecular language, in which ncRNAs have emerged as key regulatory nodes. Understanding the mechanisms by which these ncRNAs transmit information across different cellular compartments or modulate intrinsic cellular functions is fundamental to deciphering the logic of immune regulation and tumor progression. This section delves into the principal molecular strategies—extracellular vesicle trafficking, competitive endogenous RNA networks, and transcriptional/epigenetic regulation—that enable ncRNAs to fine-tune the three core regulatory axes detailed in subsequent sections.

### 2.1. Extracellular Vesicles: The Messengers of ncRNA Communication

Extracellular vesicles (EVs), particularly exosomes, have been established as pivotal conduits for the intercellular transfer of ncRNAs, facilitating a critical channel of communication between tumor, stromal, and immune cells [16]. These lipid bilayer-enclosed nanoparticles are loaded with selective molecular cargo, including proteins, lipids, and various ncRNA species, and are released into the extracellular space upon fusion of multivesicular bodies with the plasma membrane [17]. The bioactivity of EV-derived ncRNAs is protected from enzymatic degradation during transit, enabling them to exert functional effects upon delivery to recipient cells [16,17].

Tumor cells and CAFs exploit this pathway to disseminate oncogenic signals and sculpt an immunosuppressive niche. For instance, CAF-derived exosomes carry specific lncRNAs, such as the EV-derived lncRNA PWAR6, which can be internalized by NK cells [18]. Within the NK cell cytoplasm, PWAR6 functions as a competitive endogenous RNA to sequester miRNAs, leading to the dysregulation of target genes involved in cellular metabolism and ultimately repressing NK cell cytotoxicity. Similarly, tumor-derived exosomes deliver the lncRNA H19, which can modulate gene expression programs in macrophages, skewing them towards a pro-tumorigenic M2 phenotype [19,20]. Beyond exosomes, other EV subtypes, including microvesicles that bud directly from the plasma membrane, contribute to this ncRNA-rich informational network [21]. This vesicular trafficking represents a global mechanism for the systemic rewiring of the TME, establishing a pro-metastatic niche and conferring resistance to therapies, including those targeting immune checkpoints.

### 2.2. The Competitive Endogenous RNA Network

The ceRNA hypothesis presents a paradigm-shifting framework for understanding post-transcriptional regulation, positing that diverse RNA transcripts communicate through shared miRNA response elements (MREs) [22]. Within this intricate regulatory lattice, endogenous lncRNAs and circRNAs function as molecular sponges or decoys, competitively binding to miRNAs and thereby preventing them from interacting with their canonical mRNA targets [23]. This sequestration effectively derepresses the targeted mRNAs, leading to altered protein expression that can profoundly impact NK cell development, recruitment, and effector function.

CircRNAs, with their covalently closed continuous loop structure that confers exceptional stability, are frequently cited as potent ceRNAs. A canonical example is circRNA CDR1as, which contains multiple binding sites for miR-7. In the context of the TME, elevated levels of endogenous CDR1as can sponge miR-7, leading to the increased expression of miR-7 target genes that may influence growth factor signaling and immune cell activity [24]. Likewise, specific lncRNAs can act as ceRNAs to modulate immune responses. The endogenous lncRNA NKILA serves as a compelling example; it can sequester miRNAs that target immunosuppressive mRNAs, resulting in the upregulation of proteins that inhibit T cell and NK cell cytotoxicity [25]. This ceRNA crosstalk creates a vast, interconnected regulatory network that allows tumor and stromal cells to fine-tune gene expression dynamically. The dysregulation of a single key ncRNA node within this network can have cascading effects, ultimately reprogramming the immune landscape to favor tumor progression.

While the ceRNA hypothesis presents an elegant regulatory framework, its physiological applicability necessitates rigorous stoichiometric evaluation [26]. Absolute RNA quantification and mathematical modeling consistently indicate that most endogenous lncRNAs and circRNAs are maintained at low cellular copy numbers, often dwarfed by the robust intracellular abundance of their targeted miRNAs [27]. For a transcript to function as a biologically relevant miRNA sponge, the cumulative density of its available MREs, coupled with a high binding affinity, must achieve a threshold sufficient to functionally titrate the unbound miRNA pool [28]. Consequently, barring instances of pronounced transcriptional amplification or restricted subcellular compartmentalization that locally enriches MRE concentration, the competitive derepression of mRNA targets by sequestering miRNAs is stoichiometrically constrained under basal conditions [29]. Consequently, while many studies rely on ectopic overexpression models to demonstrate robust ceRNA crosstalk, these experimental systems often achieve transcript abundances that far exceed physiological levels. Establishing authentic regulatory relevance within the TME requires precise validation of absolute transcript abundances and specific binding affinities, thereby mitigating the experimental artifacts frequently introduced by supraphysiological overexpression models.

### 2.3. Transcriptional and Epigenetic Regulation

Beyond their roles in post-transcriptional control, endogenous lncRNAs exert profound influence over gene expression programs by directly modulating transcriptional and epigenetic states. These ncRNAs can guide chromatin-modifying complexes to specific genomic loci or interact with transcription factors to alter their activity, thereby enacting long-lasting changes in cell identity and function—a mechanism of particular importance for defining NK cell phenotypes in the plastic and dynamic TME [29].

A seminal example is the lncRNA HOTAIR, which acts as a molecular scaffold for the Polycomb Repressive Complex 2 (PRC2) and the LSD1/CoREST/REST complex [30]. By recruiting these repressive complexes to specific gene promoters, HOTAIR catalyzes histone H3 lysine 27 trimethylation (H3K27me3) and demethylation of H3K9me2 [31,32], effectively silencing a suite of tumor suppressor and immunomodulatory genes. In immune cells, this mechanism can be co-opted to enforce dysfunctional states. For instance, the endogenous lncRNA MALAT1 can interact with transcription factors involved in the NF-κB pathway, a central regulatory node of inflammation and immune activation [33]. By modulating the nuclear translocation or DNA-binding affinity of these factors, MALAT1 can fine-tune the expression of key cytokines, chemokines, and surface receptors that dictate the crosstalk between stromal and immune compartments [34]. This capacity to directly rewire the epigenetic and transcriptional landscape establishes lncRNAs as powerful molecular modulators that can lock NK cells into tolerant or exhausted states, thereby subverting antitumor immunity.

These core mechanisms provide the molecular basis for the bidirectional crosstalk between NK cells and other TME components, as well as the intrinsic regulation of NK cells themselves, which will be elaborated in the following sections.

## 3. Mechanisms of ncRNA-Regulated Interactions Between NK Cells and the Tumor Microenvironment

The TME is a critical niche for tumor initiation, progression, and metastasis, consisting of a complex interaction network involving tumor cells, stromal cells, immune cells, and extracellular components. NK cells, function as core effectors of the innate immune system, recognizing and eliminating malignant cells without prior sensitization. However, the immunosuppressive properties of the TME frequently induce NK cell dysfunction. Emerging evidence suggests that ncRNAs, including lncRNAs, circRNAs, and miRNAs, may act as key molecular nodes regulating both intercellular crosstalk and intracellular functional homeostasis. In the crosstalk between NK cells and the TME, ncRNAs are hypothesized to participate in a multi-level regulatory network: it not only includes the extrinsic regulation of NK cells by ncRNAs secreted by other cells in the TME, but also covers the reverse regulation of various TME components by ncRNAs secreted by NK cells themselves. Meanwhile, endogenous ncRNAs in NK cells appear to finely regulate their own functions through intrinsic networks. This chapter systematically integrates the above three regulatory dimensions to comprehensively elaborate on the proposed molecular mechanisms of ncRNA-mediated regulation of NK cell-TME interactions, providing a theoretical basis for developing novel ncRNA-based antitumor immunotherapeutic strategies. This section systematically elucidates the mechanisms of ncRNA-mediated regulation across three distinct dimensions: the extrinsic modulation of NK cells by secreted and EV-derived ncRNAs from TME-resident cells; the reciprocal regulation of TME components by NK cell-derived ncRNAs; and the intrinsic regulation of NK cell biology by endogenous ncRNAs.

### 3.1. Regulation of NK Cells by ncRNAs Derived from Other TME Cells

Numerous cells within the TME, such as tumor cells, CAFs, immune cells, and endothelial cells, secrete diverse ncRNAs. These ncRNAs are proposed to target NK cells through extracellular vesicles or direct release, potentially influencing NK cell antitumor function across multiple dimensions, including recruitment, activation, cytotoxicity, and apoptosis. Below, we elaborate on these regulatory effects by classifying the source cells, with a focus on the functional impacts of ncRNAs on NK cells, just as the inhibition or modulation of core biological processes.

Mechanisms by which TME-derived ncRNAs suppress NK cells can be strictly delineated into two modalities: indirect microenvironmental modulation and direct cellular targeting. Indirect effects occur when endogenous ncRNAs within tumor or stromal cells alter the surface ligands or chemokine gradients required for NK cell recruitment and recognition. Direct effects occur when ncRNAs are packaged into EVs, transferred into the extracellular space, and subsequently internalized by recipient NK cells, directly altering their intracellular signaling cascades and metabolic fitness.

#### 3.1.1. Tumor Cell-Derived ncRNAs

**Figure 1:**
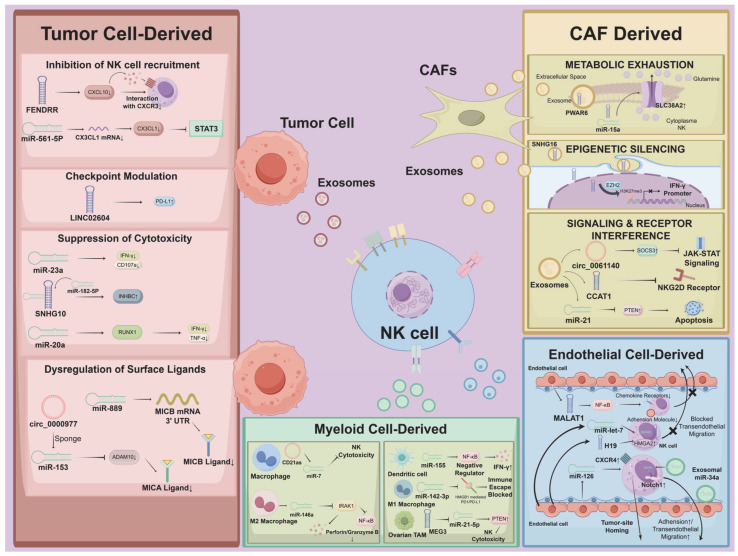
Mechanisms of ncRNA-mediated NK cell suppression in the tumor microenvironment. Tumor cells employ ncRNAs to inhibit recruitment (FENDRR, miR-561-5p), modulate checkpoints (LINC02604), suppress cytotoxicity (miR-23a, miR-20a, and SNHG10 sponging miR-182-5p), and dysregulate surface ligands (miR-889 and circ_0000977 sponging miR-153). CAFs drive metabolic exhaustion (PWAR6, miR-15a) and epigenetic silencing (SNHG16), while interfering with NK signaling receptors (circ_0061140, CCAT1, miR-21). Myeloid cells, including various macrophage subsets and dendritic cells, impair NK cytotoxicity and recognition via a suite of ncRNAs (miR-221, miR-145a, miR-146a, miR-155, miR-142-3p, MEG3). Furthermore, endothelial cells block NK cell migration and homing through specific lncRNA/miRNA axes (MALAT1/let-7, H19, miR-126) and exosomal miR-34a. These diverse exosomal and intracellular ncRNA pathways collectively facilitate tumor immune evasion. (Solid arrows indicate general stimulatory effects, while bar-headed arrows represent inhibitory effects. Arrows are directed from the upstream regulatory factor to its downstream target, and the thickness of each arrow reflects the relative strength or biological significance of the depicted interaction.).

Tumor cell-derived ncRNAs systematically have been associated with the impairment of NK cell antitumor immunity by employing evasion strategies that compromises recruitment, recognition, and effector function (Figure 1 and Table 1).

Indirectly, tumor endogenous ncRNAs disrupt chemotactic axes. Direct in vitro functional validation has shown that upregulated endogenous FENDRR suppresses CXCL10 expression in tumor cells, impairing the interaction with CXCR3 on NK cells and reducing tumor infiltration [35,36]. Similarly, in hepatocellular carcinoma (HCC), the upregulation of endogenous miR-561-5p has been shown in vitro to target the mRNA of CX3CL1 within tumor cells, suppressing its secretion. This blockade not only hinders the recruitment of CX3CR1^+^ NK cells but also prevents their STAT3 signaling-dependent activation, facilitating pulmonary metastasis [37]. Also, tumors further evade surveillance by dysregulating the surface ligands necessary for NK cell activation. In vitro evidence indicates that in HCC, miR-889 confers resistance to NK cell-mediated lysis by directly targeting the 3′-untranslated region (3′-UTR) of MICB mRNA, consequently downregulating the surface expression of this critical NKG2D ligand [38]. Alternatively, recognition can be impaired through ligand shedding; in the hypoxic microenvironment of pancreatic cancer, indirect functional evidence suggests that upregulated circ_0000977 functions as a sponge for miR-153. This derepresses HIF1A-mediated ADAM10 expression, which subsequently promotes the shedding of the activating ligand MICA from the tumor surface, enabling immune escape [39]. Furthermore, bioinformatic associations in clinical bladder cancer samples suggest that the m6A-related endogenous lncRNA LINC02604 correlates with upregulated PD-L1 expression. Notably, this high-risk environment is characterized by a high proportion of activated NK cells rather than resting ones, suggesting that the tumor escapes clearance primarily through PD-L1-mediated checkpoint inhibition despite the presence of infiltrating effectors, indicating a potential mechanism for checkpoint-mediated evasion [40]. Conversely, tumor cells directly suppress NK cell cytotoxicity via verified exosomal transfer. Exosomal SNHG10 derived from colorectal cancer cells is internalized by NK cells, where it sponges endogenous miR-182-5p to upregulate INHBC expression, thereby dampening cytotoxicity [41]. Similarly, exosomes from cervical cancer cells deliver miR-20a directly to NK cells, where it targets the hematopoietic transcription factor RUNX1, suppressing proliferation and the secretion of IFN-γ and TNF-α [42]. Additionally, direct in vitro studies indicate that secreted miR-23a impairs cytotoxicity by reducing CD107a degranulation in activated NK cells [43].

#### 3.1.2. Cancer-Associated Fibroblast-Derived ncRNAs

CAFs exert profound effects on NK cell function primarily through EV-mediated direct targeting that induces metabolic exhaustion, suppressing activating receptors, and suppresses cytokine secretion (Figure 1). Direct in vitro exosomal transfer models demonstrate that myCAF-derived EVs deliver the lncRNA PWAR6 into NK cells. Inside the NK cell, functional assays suggest that PWAR6 acts as a ceRNA to sequester endogenous miR-15a, leading to upregulation of the glutamine transporter SLC38A2, triggering glutamine competition in the TME and rapidly inducing NK cell metabolic exhaustion [18]. To inhibit cytokine secretion, breast cancer CAF-derived EVs deliver SNHG16 to NK cells, where it interacts with EZH2 to induce repressive H3K27me3 epigenetic modifications at the IFNG promoter, shutting down IFN-γ production [44,45]. Indirectly, endogenous CAF lncRNAs also play a role; CAF-derived lncRNA CCAT1 has been shown in vitro to function as a ceRNA to suppress the expression of NKG2D—a key activating receptor on NK cells—thereby reducing their ability to recognize and eliminate tumor cells [46], while endogenous hsa_circ_0061140 in colorectal CAFs sponges miR-338-3p to upregulate SOCS3, inhibiting the broader inflammatory networks that support NK cell activation [43].

#### 3.1.3. Myeloid Cell-Derived ncRNAs

As shown in Figure 1 and Table 1, myeloid-derived ncRNAs highlight the functional plasticity of immune crosstalk, offering both direct and indirect regulatory inputs. Regarding direct targeting, macrophage-derived EVs deliver CDR1as to NK cells, where it acts as a potent ceRNA to sequester endogenous miR-7, altering signaling pathways to skew the immune response toward tumor tolerance [47,48]. M2-polarized macrophages secrete exosomal miR-146a, which targets IRAK1 in recipient NK cells to impair NF-κB-dependent perforin and granzyme B expression [49,50]. In contrast, M1-polarized macrophages secrete miR-142-3p, which targets the HMGB1/PD-L1 pathway in tumor cells, indirectly enhancing NK cell-mediated antitumor immunity [51]; In ovarian cancer, restoring the lncRNA MEG3 in tumor-associated macrophages (TAMs) promotes the secretion of exosomal MEG3, which directly internalizes into NK cells, repressing miR-21-5p and enhancing cytotoxicity [52]. Furthermore, verified exosomal transfer from mature dendritic cells (DCs) delivers miR-155 to NK cells, enhancing NF-κB signaling and IFN-γ secretion [53,54].

While these EV-mediated ceRNA networks present compelling mechanisms for intercellular immunosuppression, their quantitative feasibility in vivo warrants careful interpretation [26]. The functional outcome of an internalized EV-derived ceRNA is strictly governed by the stoichiometric ratio of the delivered sponge relative to the vast endogenous miRNA pool within the recipient NK cell [27,28]. Many foundational studies validating these specific axes currently rely on in vitro EV co-culture systems or exogenous overexpression models, which may not accurately reflect the physiological transcript abundance transferred within the complex, diffusion-limited architecture of the TME [16,27]. Therefore, defining the authentic target occupancy and binding affinity of these exogenous transcripts at physiological concentrations remains a critical frontier for validating EV-mediated ceRNA networks in human malignancies [29].

#### 3.1.4. Endothelial Cell-Derived ncRNAs

Vascular endothelial cells regulate NK cell recruitment and infiltration primarily through indirect mechanisms. Bioinformatic and in vitro validations reveal that endothelial-expressed endogenous lncRNA MALAT1 interacts with transcription factors in the NF-κB pathway, suppressing the expression of adhesion molecules required for NK cell transendothelial migration [34]. Conversely, endothelial endogenous miR-126 enhances the expression of homing-associated chemokines [19,55]. Regarding direct EV communication, in vitro studies suggest that gastric tumor endothelial cells secrete exosomal H19, which is internalized by NK cells and acts as a ceRNA to sequester endogenous miR-let-7, upregulating HMGA2 and inhibiting NK cell migration across the endothelial barrier [19,41,55]. However, delivering exogenous miR-34a to lung cancer endothelial cells has been shown in vitro to promote the secretion of exosomal miR-34a, which directly targets Notch1 in NK cells to enhance their adhesion to endothelial cells and transendothelial migration, whose detailed mechanisms are indicated in Figure 1 and Table 1 [56,57].

When interpreting the immunomodulatory effects of secreted and EV-derived ncRNAs, it is critical to recognize their inherent pleiotropy. Transcripts such as MALAT1, GAS5, miR-155, and miR-21 are frequently associated with opposing functional outcomes across different studies. This biological paradox is not contradictory; rather, it indicates that the functional consequence of an extracellular ncRNA is reliably predicted by the interplay of three key determinants: donor cell identity, EV transport mechanisms, and the recipient cell type. For instance, the immunomodulatory role of miR-155 shifts dramatically based on these variables. When the donor is a mature dendritic cell, the transport mechanism involves EVs densely co-packaged with costimulatory molecules; upon internalization by a recipient NK cell, this specific cargo provides a potent activating signal that synergizes with the co-delivered molecules [53,54]. Conversely, when the same miR-155 transcript is released by a malignant tumor cell via an immunosuppressive EV transport mechanism, it often promotes immune evasion by upregulating compensatory survival pathways in recipient lymphocytes. Mechanistically, even when the donor and transport parameters are established, the ultimate functional outcome is governed by the stoichiometric availability of target mRNAs or miRNAs within the recipient cell. This intracellular profile drastically shifts depending on whether the recipient NK cell is resting, actively proliferating, or metabolically exhausted [27]. Therefore, defining an extracellular ncRNA as universally “pro-tumor” or “antitumor” oversimplifies the dynamic, state-dependent nature of TME regulatory networks; instead, their functional roles must be continuously evaluated across this complex donor–transport–recipient landscape.

**Table 1:** Regulation of NK Cells by ncRNAs Derived from Other Cells in the TME.

ncRNA Name	Type	TME Cell Type	Molecular Axis	Functional Effect	References
FENDRR	lncRNA	Various tumor cells	FENDRR/CXCL10 axis	Impairs NK cell recruitment, forming a “chemokine desert”	[35,36]
LINC02604	lncRNA	Bladder cancer cells	LINC02604/m6A/PD-L1 axis	Enriches resting NK cells and inhibits NK cell activation	[40]
H19	lncRNA	Tumor cells	H19/NK cell activation signaling axis	Weakens NK cell activation and cytotoxicity	[19,20]
SNHG10	lncRNA	Colorectal cancer cells	SNHG10/INHBC axis	Inhibits NK92-MI cell cytotoxicity and promotes CRC tumor growth	[41]
circ_0000977	circRNA	Pancreatic cancer cells (hypoxic)	circ_0000977/miR-153 axis	Weakens NK cell killing of hypoxic pancreatic cancer cells	[39,58]
miR-23a	miRNA	Hepatocellular carcinoma cells	miR-23a/ CD107a axis	Downregulates NKG2D receptor expression and impairs NK cell tumor recognition	[43]
miR-20a	miRNA	Cervical cancer cells	miR-20a/RUNX1 axis	Reduces NK cell cytotoxicity against cervical cancer cells	[42]
miR-561-5p	miRNA	Hepatocellular carcinoma cells	miR-561-5p/CX3CL1-CX3CR1-STAT3 axis	Inhibits NK cell infiltration and promotes HCC tumor metastasis	[37]
miR-889	miRNA	Hepatocellular carcinoma cells	miR-889/MICB axis	Reduces NK cell cytotoxicity against HCC cells	[38]
PWAR6	lncRNA	Breast cancer CAFs	PWAR6/miR-15a/SLC38A2 axis	Triggers glutamine competition, induces NK cell metabolic exhaustion and functional impairment	[18]
CCAT1	lncRNA	CAFs	CCAT1/NKG2D axis	Reduces NK cell tumor recognition and killing ability	[46]
SNHG16	lncRNA	Breast cancer CAFs	SNHG16/EZH2/IFN-γ promoter H3K27me3 axis	Inhibits IFN-γ secretion by NK cells and reduces cytotoxicity	[44,45]
hsa_circ_0061140	circRNA	Colorectal cancer CAFs	hsa_circ_0061140/miR-338-3p/SOCS3-JAK-STAT axis	Upregulates SOCS3, inhibits the JAK-STAT pathway, and hinders NK cell activation and proliferation	[59]
MEG3	lncRNA	Ovarian cancer TAMs	MEG3/miR-21-5p/PTEN axis	Upregulates PTEN in NK cells and enhances cytotoxicity against ovarian cancer cells	[52]
CDR1as	circRNA	Macrophages	CDR1as/miR-7 axis	Upregulates miR-7 target genes, inhibits NK cell cytotoxicity, and favors tumor tolerance	[47,48]
miR-155	miRNA	Dendritic cells	miR-155/NF-κB negative regulator axis	Enhances IFN-γ secretion and cytotoxicity of NK cells	[53,54]
miR-146a	miRNA	Pancreatic M2-type macrophages	miR-146a/IRAK1-NF-κB axis	Inhibits NF-κB activation, reduces perforin/granzyme B secretion, and suppresses NK cell function	[49,50]
miR-142-3p	miRNA	M1-type macrophages	miR-142-3p/HMGB1-PD1/PD-L1 axis	Indirectly enhances NK cell killing of glioblastoma (inhibits tumor immune escape)	[51]
MALAT1	lncRNA	Endothelial cells	MALAT1/NF-κB-adhesion molecule axis	Reduces NK cell transendothelial migration and tumor infiltration	[34]
H19	lncRNA	Gastric tumor endothelial cells	H19/let-7/HMGA2 axis	Upregulates HMGA2 and inhibits NK cell migration across the endothelial barrier	[55]
miR-126	miRNA	Endothelial cells	miR-126/CXCR4 regulator axis	Enhances NK cell homing to tumor sites	[60,61]
miR-34a	miRNA	Lung cancer endothelial cells	miR-34a/Notch1 axis	Enhances NK cell adhesion to endothelial cells and promotes tumor infiltration	[56,57]

### 3.2. Regulation of TME Components by NK Cell-Derived ncRNAs

**Figure 2:**
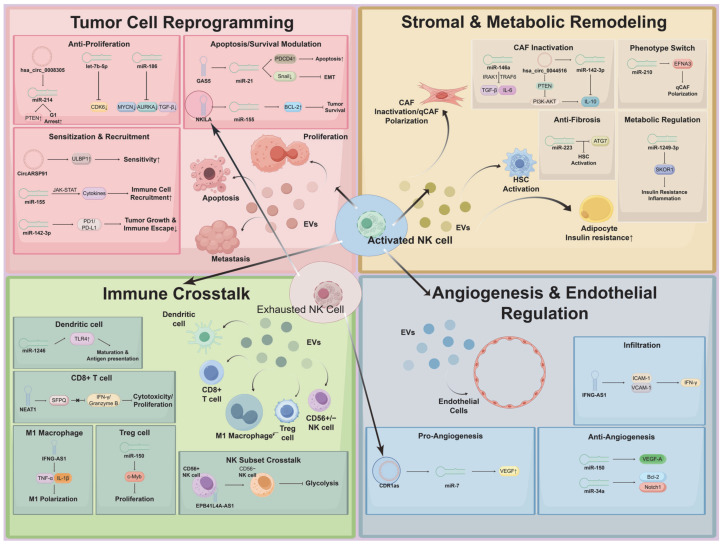
Modulation of TME Components by NK Cell-Derived ncRNAs. This figure illustrates the multifaceted roles of ncRNAs secreted by NK cells in remodeling the tumor microenvironment (TME). Depending on the activation or exhaustion state of the parent NK cell, these ncRNAs can directly reprogram tumor cells, modulate stromal and metabolic components, regulate angiogenesis, and orchestrate immune crosstalk, ultimately influencing either antitumor immunity or immune escape. (Solid arrows indicate general stimulatory effects, while bar-headed arrows represent inhibitory effects. Arrows are directed from the upstream regulatory factor to its downstream target, and the thickness of each arrow reflects the relative strength or biological significance of the depicted interaction.).

Similar to TME-resident cells, NK cells actively secrete ncRNAs via EVs to reciprocally modulate the tumor microenvironment. These NK cell-derived ncRNAs can be broadly categorized into transcripts that directly target tumor cells to inhibit proliferation and survival, and those that modulate stromal and other immune cells to reshape the TME (Figure 2 and Table 2). Importantly, the cargo of NK cell-derived EVs dynamically shifts depending on whether the NK cell is in an activated, resting, or exhausted phenotypic state.

#### 3.2.1. Regulation of Tumor Cell Biology

Direct in vitro functional validations demonstrate that EVs from activated NK cells carry tumor-suppressive cargo. NK cell-derived circRNA hsa_circ_0008305 has been shown in vitro to target miR-214 in tumor cells, upregulating PTEN and inducing G1 phase cell cycle arrest [50,62]; NK cell-secreted lncRNA GAS5 is proposed to act as a ceRNA to sponge miR-21 in breast cancer cells, associated with de-repressed PDCD4 expression and promoted apoptosis, as well as downregulated Snail to inhibit epithelial–mesenchymal transition (EMT) [63]; and exosomal let-7b-5p from NK cells targets the cell cycle regulator CDK6 in pancreatic cancer cells to control proliferation [64]. as for inhibiting angiogenesis and metastasis, NK cell-derived miR-223 targets VEGFR2 in tumor cells, inhibiting tumor angiogenesis and reducing metastatic potential [65]; exosomal miR-186 from NK cells is reported to directly target MYCN, AURKA, and the TGF-β pathway, restraining neuroblastoma growth, dissemination, and immune escape [43,66]. NK cell-secreted circARSP91 upregulates ULBP1 (a key activating ligand for NK cells) in HCC cells, sensitizing them to NK cell cytotoxicity [67], so as to enhance tumor cell sensitivity to NK cell cytotoxicity. With regard to immune-related pathway modulation, indirect functional evidence shows that miR-155 secreted by activated NK cells targets SOCS1 in melanoma cells. This targeting event correlates with activation of the JAK-STAT pathway and upregulated expression of pro-inflammatory cytokines, which in turn promotes the recruitment of more immune cells [68]; miR-142-3p from NK cells targets the HMGB1-mediated PD1/PD-L1 pathway in glioblastoma cells, inhibiting tumor growth and immune escape [51]. Additionally, NK cell-derived lncRNA NKILA is delivered to tumor cells via EVs, acting as a ceRNA to sequester miR-155, upregulating the anti-apoptotic protein BCL-2 and inhibiting tumor cell apoptosis [25]. This critical mechanism illustrates how the TME subverts NK cell communication pathways, forcing exhausted immune effectors to secrete pro-tumorigenic survival factors.

#### 3.2.2. Stromal and Metabolic Regulation

Beyond direct tumor cell targeting, NK cell-derived ncRNAs modulate the activation, proliferation, and cytokine secretion of various TME cells, alleviating stromal-mediated immunosuppression. Regarding CAF activation inhibition, in vitro studies indicate NK cell-derived miR-146a targets IRAK1 and TRAF6—key components of the NF-κB pathway—in CAFs, suppressing CAF activation and reducing the secretion of immunosuppressive cytokines like IL-6 and TGF-β [46]; Meanwhile, NK cell-derived circRNA hsa_circ_0044516 is proposed to sponge miR-142-3p in CRC-derived CAFs, upregulating PTEN and inhibiting the PI3K-AKT pathway to restrain CAF activation and IL-10 secretion [69]. For suppressing CAF proliferation and migration, NK cell-secreted lncRNA GAS5 is hypothesized to function as a ceRNA to sequester miR-10a in CAFs, de-repressing PTEN expression and curbing CAF proliferation and migration [62]. In terms of inducing CAF phenotype switching, miR-210 released by hypoxic NK cells targets EFNA3 in CAFs [70], prompting their polarization toward a quiescent phenotype (qCAF) and reducing the secretion of pro-tumorigenic factors [71]. NK cell-derived ncRNAs are also suggested to modulate TME metabolic microenvironments: exosomal miR-1249-3p from NK cells is shown in vivo to target SKOR1 in adipocytes and hepatocytes, improving insulin sensitivity and mitigating inflammation, which indirectly impacts angiogenesis by regulating the TME metabolic landscape [72]. Moreover, they are reported to exhibit anti-fibrotic effects—NK cell-derived miR-223 targets VEGFR2 in tumor cells, thereby inhibiting tumor angiogenesis and reducing metastatic potential [65].

#### 3.2.3. Regulation of Vascular Endothelial Cells and Angiogenesis

NK cell-derived ncRNAs further regulate tumor angiogenesis by targeting endothelial cells, with pro- and anti-angiogenic effects balancing to influence TME vascular density. Focusing on pro-angiogenic effects, in vitro functional evidence shows NK cell-derived circRNA CDR1as targets miR-7 in endothelial cells, upregulating VEGF and promoting endothelial cell proliferation and tube formation [47,48]. In contrast, their anti-angiogenic effects are mediated by specific miRNAs: NK cell-secreted miR-34a targets Bcl-2 and Notch1 in endothelial cells, suppressing proliferation and tube formation [56,57]. Additionally, these ncRNAs facilitate immune cell infiltration: NK cell-derived lncRNA IFNG-AS1 is correlated with enhanced the expression of IFN-γ-inducible genes such as ICAM-1 and VCAM-1 in endothelial cells, potentially promoting immune cell adhesion and infiltration into the tumor parenchyma [73].

#### 3.2.4. Regulation of Other Immune Cells

NK cell-derived ncRNAs also fine-tune the function of other immune cells in the TME, coordinating the antitumor immune response across multiple cell types. For macrophage regulation, in vitro, NK cell-derived lncRNA IFNG-AS1 promotes M1 polarization of macrophages by enhancing the expression of pro-inflammatory cytokines such as TNF-α and IL-1β [74]. In terms of Treg cell inhibition, NK cell-secreted miR-150 is shown in vitro to target c-Myb in Treg cells, effectively suppressing their proliferation [75]. For DC maturation promotion, in vitro evidence indicates NK cell-derived miR-1246 upregulates TLR4 expression in DCs, facilitating their maturation and antigen presentation to T cells to amplify the immune response [76,77]. When it comes to enhancing CD8+ T cell function, NK cell-secreted lncRNA NEAT1 is reported to interact with the transcriptional repressor SFPQ in CD8+ T cells, relieving the suppression of IFN-γ and granzyme B expression and thereby boosting CD8+ T cell cytotoxicity and proliferation [78]. Additionally, regulation mechanisms are conducted on NK cell subset crosstalk: CD56+ NK cells secrete exosomal lncRNA EPB41L4A-AS1, which has been shown in vitro to be transferred to CD56− NK cells and inhibits their glycolysis, ultimately contributing to neuroblastoma immune escape [79].

The secretory profile of NK cells provides a stark example of how cellular phenotype drives ncRNA pleiotropy. The functional impact of NK cell-derived EVs—such as those containing GAS5, miR-150, or NKILA—is fundamentally governed by the activation and exhaustion state of the parent NK cell. Rather than viewing the alteration in EV cargo as a mere paradox, this shift represents a predictable outcome driven directly by the intersection of disease progression and metabolic fitness. This dynamic establishes a strict predictive rule: during early disease stages, characterized by acute immune activation and high metabolic fitness, NK cells utilize specific RNA-binding proteins to selectively sort tumor-suppressive ncRNAs into EVs, thereby suppressing tumor angiogenesis and limiting cancer-associated fibroblast (CAF) activation [80]. Conversely, in advanced disease stages defined by chronic TME stress, severe metabolic exhaustion induces maladaptive epigenetic and transcriptomic reprogramming within the NK cell. This profound metabolic impairment actively reprograms the EV sorting machinery, dictating the release of pro-tumorigenic EV cargo [81]. Consequently, a metabolically exhausted NK cell in a late-stage tumor releases EVs enriched with ncRNAs that inadvertently support tumor survival, tissue remodeling, and endothelial proliferation. This functional dichotomy highlights that NK cell-derived ncRNAs are not inherently protective but are dynamic reflections of the cell’s immunological and metabolic fitness.

**Table 2:** Effects of TME Components on the NK Cell-Derived ncRNAs.

ncRNA Name	Type	TME Target Component	Molecular Axis	Functional Effect	References
NKILA	lncRNA	Tumor cells	NKILA/miR-155/BCL-2 axis	Upregulates BCL-2, inhibits tumor cell apoptosis, and promotes survival	[25]
GAS5	lncRNA	Breast cancer cells	GAS5/miR-21/PDCD4 axis	Upregulates PDCD4, promotes tumor cell apoptosis; downregulates Snail, inhibits EMT	[63]
IFNG-AS1	lncRNA	Endothelial cells	IFNG-AS1/IFN-γ-inducible gene (ICAM-1/VCAM-1) axis	Promotes adhesion between immune cells and endothelial cells, facilitating tumor infiltration	[73,74]
circARSP91	circRNA	Hepatocellular carcinoma cells	circARSP91/ULBP1 axis	Enhances the killing activity of NK cells against HCC cells	[67]
hsa_circ_0008305	circRNA	Tumor cells	hsa_circ_0008305/miR-214/PTEN axis	Induces G1 phase arrest in tumor cells and inhibits proliferation	[62,82]
CDR1as	circRNA	Endothelial cells	CDR1as/miR-7/VEGF axis	Promotes endothelial cell proliferation and tube formation, enhances angiogenesis	[45]
miR-1246	miRNA	Dendritic cells	miR-1246/TLR4 axis	Upregulates TLR4, promotes DC maturation and antigen presentation	[76,77]
miR-223	miRNA	Tumor cells	miR-223/VEGFR2 axis	Inhibits tumor angiogenesis and reduces metastatic potential	[65]
miR-223	miRNA	Hepatic stellate cells	miR-223/ATG7 axis	Inhibits TGF-β-induced HSC activation and alleviates liver fibrosis	[83]
miR-155	miRNA	Melanoma cells	miR-155/SOCS1-JAK-STAT axis	Enhances the expression of pro-inflammatory cytokines in tumor cells and recruits more immune cells	[68]
miR-186	miRNA	Neuroblastoma cells	miR-186/MYCN-AURKA-TGF-β axis	Inhibits tumor growth, dissemination, and immune escape	[66]
let-7b-5p	miRNA	Pancreatic cancer cells	let-7b-5p/CDK6 axis	Inhibits pancreatic cancer cell proliferation and controls tumor growth	[64]
miR-142-3p	miRNA	Glioblastoma cells	miR-142-3p/HMGB1-PD1/PD-L1 axis	Inhibits tumor growth and immune escape, enhances NK cell-mediated antitumor immunity	[51]
miR-34a	miRNA	Endothelial cells	miR-34a/Bcl-2/Notch1 axis	Inhibits endothelial cell proliferation and tube formation, suppresses tumor angiogenesis	[57]
miR-1249-3p	miRNA	Adipocytes/Hepatocytes	miR-1249-3p/SKOR1 axis	Improves the metabolic microenvironment of TME (alleviates inflammation)	[72]
miR-146a	miRNA	CAFs	miR-146a/IRAK1-TRAF6-NF-κB axis	Inhibits CAF activation, reduces IL-6/TGF-β secretion and collagen deposition	[46]
miR-210	miRNA	CAFs (hypoxic conditions)	miR-210/EFNA3 axis	Promotes CAF polarization toward a quiescent phenotype (qCAF) and reduces the secretion of pro-tumorigenic factors	[70,71]
NEAT1	lncRNA	CD8+ T cells	NEAT1/SFPQ/IFN-γ-granzyme B axis	Enhances CD8+ T cell proliferation and cytotoxicity	[78,84]
EPB41L4A-AS1	lncRNA	CD56dim NK cells	EPB41L4A-AS1/glycolysis axis	Induces neuroblastoma immune escape (impairs NK cell subset function)	[79]
hsa_circ_0044516	CircRNA	CAFs in CRC	hsa_circ_0044516/miR-142-3p/PTEN/ PI3K-AKT axis	suppressing CAF activation and the secretion of IL-10	[69]

### 3.3. Intrinsic Regulation of NK Cells by Endogenous ncRNAs

**Figure 3:**
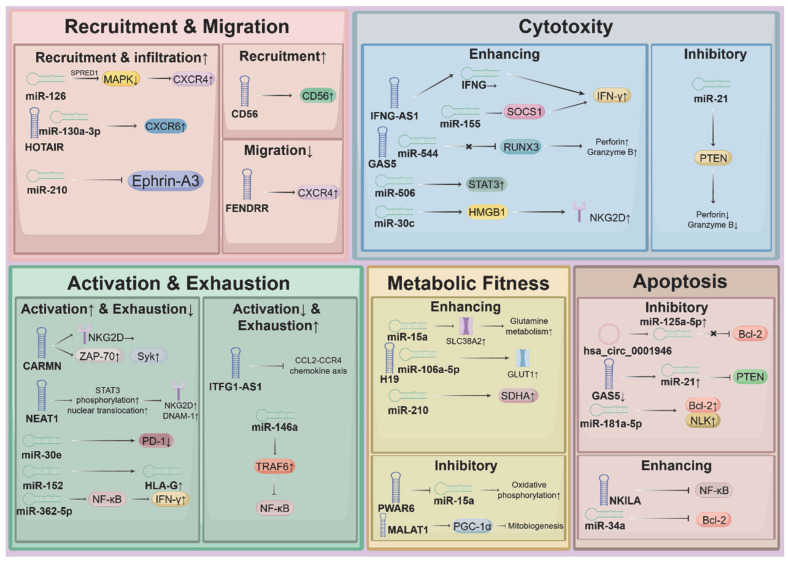
Intrinsic Modulation of NK Cells by Endogenous ncRNAs. Endogenous ncRNAs within NK cells precisely orchestrate their biological lifecycle and effector functions: regulating recruitment and migration by enhancing chemotaxis via miR-126 (targeting SPRED1/MAPK) and the HOTAIR/miR-130a-3p axis, while FENDRR acts as a negative regulator of migration; balancing cytotoxicity through the enhancing effects of IFNG-AS1 and miR-155 on IFN-γ production and the GAS5/miR-544/RUNX3 axis on perforin/granzyme B expression, which are counteracted by the inhibitory miR-21/PTEN axis; modulating activation and exhaustion by stabilizing activating receptors (NKG2D, DNAM-1) via CARMN and NEAT1 and suppressing PD-1 via miR-30e, whereas ITFG1-AS1 and miR-146a drive exhaustion phenotypes; controlling metabolic fitness by promoting nutrient uptake via miR-15a (glutamine) and H19 (glucose), while PWAR6 and MALAT1 induce metabolic impairment by suppressing mitochondrial biogenesis; and determining cell fate through pro-survival networks involving circ_0001946 and GAS5 that inhibit apoptosis, in contrast to the pro-apoptotic effects mediated by NKILA and miR-34a. (Solid arrows indicate general stimulatory effects, while bar-headed arrows represent inhibitory effects. Arrows are directed from the upstream regulatory factor to its downstream target, and the thickness of each arrow reflects the relative strength or biological significance of the depicted interaction.).

This axis strictly focuses on endogenous ncRNAs—transcripts produced and retained within the NK cell itself—that intracellularly govern essential biological programs across their dynamic spatiotemporal lifecycle, ranging from initial development and maturation to localized effector functions within the TME (Figure 3 and Table 3). Before exerting any antitumor activity, NK cells must undergo proper development and maturation, processes that are fundamentally architected by endogenous ncRNAs. LncRNAs participate early in this developmental trajectory; for example, lnc-CD56 upregulates CD56 molecule expression, preserving NK cell phenotypic characteristics and promoting their differentiation from CD34+ hematopoietic stem cells [85]. Subsequently, the transition from immature, cytokine-producing CD56^+^ NK cells to highly cytotoxic CD56^−^ NK cells is tightly governed by specific ncRNA signatures. For instance, miR-150 acts as a master regulator driving this terminal maturation process, and its absence severely impairs the generation of mature peripheral NK cells [75]. Similarly, the expression of miR-146a-5p fluctuates across different maturation stages, critically modulating the expression of killer cell immunoglobulin-like receptors and guiding the acquisition of functional competence [77]. Once a properly matured and functionally competent pool of NK cells is established, other intrinsic ncRNA networks subsequently dictate their recruitment, activation, cytotoxicity, and metabolic fitness within the hostile TME, as detailed below.

#### 3.3.1. Regulation of NK Cell Recruitment and Infiltration

Endogenous ncRNAs regulate NK cell homing by modulating intracellular signaling cascades that control the expression of chemokine receptors. Direct in vitro functional validation shows endogenous miR-126 enhances CXCR4 expression by suppressing SPRED1, promoting NK cell recruitment [86]. Indirect functional evidence indicates endogenous HOTAIR acts as an intracellular ceRNA to sequester miR-130a-3p, upregulating CXCR6 expression [87]; and miR-210, which is induced in hypoxic NK cells, is shown in vitro to target Ephrin-A3 to strengthen NK cell adhesion to endothelial cells and transendothelial migration into hypoxic tumor regions [71]. Beyond that, certain ncRNAs inhibit migration when dysregulated—lncRNA FENDRR is reported to positively modulate CXCR4 expression, and its dysregulation impairs NK cell migration toward CXCL12-expressing tumors [35].

#### 3.3.2. Regulation of NK Cell Activation and Exhaustion

Endogenous ncRNAs balance NK cell activation and exhaustion, with activating regulators promoting NK cell function and delaying exhaustion, while inhibitory regulators induce exhaustion. On the activating side, in vitro assays demonstrate lncRNA CARMN stabilizes NKG2D expression and enhances downstream signaling molecules, such as ZAP70 and Syk, to boost NK cell activation [88]; lncRNA NEAT1 is reported to interact with STAT3, promoting its phosphorylation and nuclear translocation to upregulate activating receptors, including NKG2D and DNAM-1 [78]; in vitro evidence shows miR-30e targets PD-1, reducing its expression and relieving NK cell exhaustion [89]; miR-152 targets HLA-G mRNA to enhance tumor recognition and killing [90]; and miR-362-5p sponges CYLD, activating the NF-κB pathway and augmenting cytotoxicity and IFN-γ secretion [91,92]. By contrast, bioinformatic associations combined with in vitro functional assays M7G-related lncRNA ITFG1-AS1 dysregulates the CCL2-CCR4 chemokine axis, inhibiting NK cell activation by limiting “stress signal” detection [67,93]; miR-146a, which is upregulated in exhausted NK cells, is shown to target TRAF6 to inhibit NF-κB activation and accelerate exhaustion [50].

#### 3.3.3. Regulation of NK Cell Cytotoxic Effector Functions

Endogenous ncRNAs modulate the cytotoxic output of NK cells by regulating the expression of perforin, granzymes, and IFN-γ, with most acting as enhancing regulators. In vitro evidence suggests that to enhance cytotoxicity, lncRNA IFNG-AS1 (NEST) facilitates an open chromatin configuration at the IFNG locus, stabilizing IFNG mRNA and amplifying IFN-γ production [73,74]; Intracellular lncRNA GAS5 is hypothesized to act as a ceRNA for miR-544, correlating with derepressed RUNX3, thus promoting the expression of perforin and granzyme B [44]; miR-155 targets SOCS1 to potentially boost IFN-γ production and cytotoxicity [53]; miR-506 is proposed to sponge STAT3 in vitro, inhibiting immunosuppressive pathways and enhancing cytotoxicity against HCC cells [94]; and miR-30c upregulates NKG2D expression by targeting HMGB1, strengthening cytolytic activity [95]. Conversely, a small number of ncRNAs inhibit cytotoxicity: miR-21, which is upregulated in dysfunctional NK cells, is reported to target PTEN to reduce the expression of perforin and granzyme B [96].

#### 3.3.4. Regulation of NK Cell Metabolic Fitness

Endogenous ncRNAs regulate NK cell metabolic homeostasis, extending beyond mere metabolic maintenance to orchestrate the dynamic metabolic adaptation required for NK cells to survive and function within the harsh, nutrient-deprived, and hypoxic TME. Metabolic adaptation represents an active survival strategy where NK cells rewire their energetic pathways in response to environmental stressors. Enhancing regulators promote this adaptive metabolic fitness, while inhibitory regulators induce metabolic exhaustion. For example, to maintain adaptive metabolic fitness, in vitro evidence suggests miR-15a targets the glutamine transporter SLC38A2, sustaining glutamine metabolism and preventing metabolic exhaustion [97]. Furthermore, indirect functional evidence indicates adaptation to glucose deprivation is facilitated by lncRNAs such as H19, which is hypothesized to act as a ceRNA to sequester miR-106a-5p, correlating with upregulated GLUT1 to potentially maximize glucose uptake and sustain glycolytic flux [55]. Because intrinsic ceRNA regulation relies on transcripts produced within the NK cell itself, the localized transcript abundance is more likely to reach the stoichiometric thresholds required for functional target occupancy compared to EV-derived transcripts [27,28]. Nevertheless, confirming these metabolic shifts requires transitioning from overexpression paradigms to quantitative transcriptomic mapping across different states of NK cell exhaustion to verify that the binding affinity and expression ratios between H19 and miR-106a-5p naturally support this competitive derepression in vivo [29,55]. Additionally, the hypoxia-induced miR-210 acts as a critical metabolic switch; by targeting SDHA—a key enzyme in the tricarboxylic acid cycle—it forces a shift from oxygen-dependent oxidative phosphorylation towards glycolysis, thereby preserving NK cell viability and basic functional capacity when oxygen is scarce [71,98]. However, when these adaptive mechanisms are ultimately overwhelmed by chronic TME stress, persistent ncRNA-mediated rewiring pushes the cells from a state of transient adaptation into irreversible metabolic exhaustion. Certain inhibitory ncRNAs drive this exhausted state by targeting mitochondrial integrity: In vitro studies show that lncRNA PWAR6 targets miR-15a, inhibiting mitochondrial function and reducing oxidative phosphorylation [11,18,99]; similarly, lncRNA MALAT1 is reported to interact with PGC-1α, suppressing mitochondrial biogenesis and severely impairing metabolic adaptability [34].

#### 3.3.5. Regulation of NK Cell Survival and Apoptosis

Endogenous ncRNAs balance NK cell survival and apoptosis, ensuring sustained antitumor immunity without uncontrolled proliferation. To promote survival and inhibit apoptosis, in vitro studies indicate circRNA hsa_circ_0001946 sponges miR-125a-5p, derepressing Bcl-2 expression and enhancing NK cell persistence in the TME [100]; lncRNA GAS5 is reported to target miR-21, regulating apoptotic pathways to support NK cell survival by upregulating PTEN expression in assumption [44]; and in vivo, miR-181a-5p targets Bcl-2 and NLK, maintaining NK cell proliferation and cytotoxicity, with its downregulation during aging contributing to NK cell dysfunction [101]. On the other hand, some ncRNAs promote apoptosis: in vitro models show lncRNA NKILA inhibits NF-κB signaling, sensitizing NK cells to activation-induced cell death [25,102]; miR-34a, which is upregulated in aging NK cells, suppresses Bcl-2 expression to promote apoptosis and shorten NK cell lifespan in vitro [103].

Ultimately, the delicate balance between survival and apoptosis dictates the long-term persistence of NK cells within tumors, which is a critical prerequisite for durable antitumor immunity. Tumor-infiltrating NK cells face a barrage of pro-apoptotic signals from the TME, including oxidative stress and FasL-expressing tumor cells. Endogenous ncRNAs serve as essential shields that prolong NK cell persistence in this hostile niche. CircRNAs, endowed with high structural stability, are particularly suited for enforcing long-term persistence. For example, hsa_circ_0001946 maintains a persistent effector pool by consistently derepressing the anti-apoptotic protein Bcl-2, thus preventing premature clearance of NK cells from the TME [100]. Additionally, the preservation of intra-tumoral longevity is heavily reliant on miR-181a-5p; its robust expression sustains continuous proliferation and cytotoxicity, whereas its age- or TME-induced downregulation precipitously shortens the NK cell lifespan and diminishes their persistence [101]. Consequently, therapeutic interventions that stabilize these pro-persistence ncRNA nodes hold substantial promise for enhancing the durability of adoptively transferred NK cells.

Within the intracellular environment of the NK cell, the functional pleiotropy of endogenous ncRNAs is not merely influenced by, but strictly dictated by, their spatiotemporal localization [104]. Rather than simply executing vastly different roles depending on broad biological contexts, the specific functional outcomes of endogenous lncRNAs, such as MALAT1 and GAS5, are reliably predicted by their precise subcellular compartmentalization. This dynamic establishes a clear mechanistic rule: cytoplasmic localization strictly predicts post-transcriptional regulation. For example, during initial cytokine-driven activation, specific lncRNAs localize to the cytoplasm to act as competitive endogenous RNAs (ceRNAs), buffering essential activation and survival factors against miRNA-mediated degradation. Conversely, as the NK cell becomes metabolically starved within the acidic and hypoxic TME, nuclear translocation dictates a fundamentally opposing fate. Under this TME stress, nuclear localization predicts profound epigenetic silencing; these same transcripts translocate to the nucleus to recruit repressive chromatin modifiers (such as EZH2 or the PRC2 complex), effectively silencing effector genes and enforcing terminal exhaustion [105]. Understanding that a single endogenous transcript can structurally switch from a cytoplasmic post-transcriptional survival factor to a nuclear epigenetic exhaustion driver underscores the absolute necessity of evaluating ncRNA function through the precise lens of dynamic cellular states and spatiotemporal regulation, rather than relying on static molecular models.

**Table 3:** Effects of Endogenous ncRNAs on the Intrinsic NK Cells.

ncRNA Name	Type	NK Cell Functional Module	Regulatory Mechanism	Functional Effect	References
FENDRR	lncRNA	Recruitment and Infiltration	FENDRR/CXCR4 axis	Promotes NK cell homing to CXCL12-positive tumor sites (dysregulation hinders migration)	[35]
HOTAIR	lncRNA	Recruitment and Infiltration	HOTAIR/miR-130a-3p/CXCR6 axis	Promotes NK cell homing to CXCL16-positive tumor sites	[87]
lnc-CD56	lncRNA	Recruitment and Infiltration	lnc-CD56/CD56 axis	Maintains NK cell phenotype and promotes differentiation from CD34+ hematopoietic stem cells	[85]
CARMN	lncRNA	Activation and Exhaustion	CARMN/NKG2D-ZAP70/Syk axis	Promotes NK cell activation and delays exhaustion	[88]
NEAT1	lncRNA	Activation and Exhaustion	NEAT1/STAT3/NKG2D-DNAM-1 axis	Upregulates NKG2D/DNAM-1 and delays NK cell exhaustion	[78]
ITFG1-AS1	lncRNA	Activation and Exhaustion	ITFG1-AS1/CCL2-CCR4 axis	Inhibits NK cell activation	[24,93]
NKILA	lncRNA	Survival and Apoptosis	NKILA/NF-κB axis	Sensitizes NK cells to activation-induced cell death	[25,102]
GAS5	lncRNA	Cytotoxicity	GAS5/miR-544/RUNX3 axis	Promotes perforin/granzyme B expression and enhances cytotoxicity	[44,63]
GAS5	lncRNA	Survival and Apoptosis	GAS5/miR-21/apoptotic pathway axis	Promotes NK cell survival	[44]
IFNG-AS1	lncRNA	Cytotoxicity	IFNG-AS1/IFNG gene chromatin-IFNG mRNA axis	Enhances IFN-γ secretion and improves cytotoxicity	[73,74]
H19	lncRNA	Metabolic Fitness	H19/miR-106a-5p/GLUT1 axis	Promotes glycolysis and enhances survival and function in nutrient-poor TME	[55]
MALAT1	lncRNA	Metabolic Fitness	MALAT1/PGC-1α/mitochondrial biogenesis axis	Inhibits mitochondrial biogenesis and impairs metabolic adaptability	[34]
PWAR6	lncRNA	Metabolic Fitness	PWAR6/miR-15a/mitochondrial function axis	Reduces oxidative phosphorylation and induces metabolic exhaustion	[99]
hsa_circ_0001946	circRNA	Survival and Apoptosis	hsa_circ_0001946/miR-125a-5p/Bcl-2 axis	Inhibits NK cell apoptosis and enhances persistence in TME	[100]
miR-126	miRNA	Recruitment and Infiltration	miR-126/SPRED1-CXCR4 axis	Promotes NK cell recruitment	[104]
miR-210	miRNA	Recruitment and Infiltration	miR-210/Ephrin-A3 axis	Enhances NK cell adhesion to endothelial cells and improves infiltration into hypoxic tumor regions	[71]
miR-30e	miRNA	Activation and Exhaustion	miR-30e/PD-1 axis	Reduces PD-1 expression and relieves exhaustion	[89]
miR-146a	miRNA	Activation and Exhaustion	miR-146a/TRAF6-NF-κB axis	Promotes NK cell exhaustion	[50]
miR-152	miRNA	Activation and Exhaustion	miR-152/HLA-G axis	Enhances NK cell tumor recognition and killing	[90]
miR-362-5p	miRNA	Activation and Exhaustion	miR-362-5p/CYLD-NF-κB axis	Enhances NK cell cytotoxicity and IFN-γ secretion	[91,92]
miR-155	miRNA	Cytotoxicity	miR-155/SOCS1/cytokine signaling axis	Improves IFN-γ secretion and cytotoxicity	[53]
miR-21	miRNA	Cytotoxicity	miR-21/PTEN/perforin-granzyme B axis	Reduces perforin/granzyme B expression and impairs cytotoxicity	[96]
miR-506	miRNA	Cytotoxicity	miR-506/STAT3/immunosuppressive pathway axis	Enhances cytotoxicity against HCC cells	[94]
miR-30c	miRNA	Cytotoxicity	miR-30c/NKG2D axis	Enhances cytolytic activity against HCC cells	[95]
miR-15a	miRNA	Metabolic Fitness	miR-15a/SLC38A2/glutamine metabolism axis	Prevents metabolic exhaustion	[97]
miR-210	miRNA	Metabolic Fitness	miR-210/SDHA/glycolytic metabolism axis	Enhances survival in hypoxic TME	[98]
miR-34a	miRNA	Survival and Apoptosis	miR-34a/BCL-2 (inhibition of anti-apoptotic BCL-2 expression)	Promotes apoptosis and shortens lifespan	[103]
miR-181a-5p	miRNA	Survival and Apoptosis	miR-181a-5p/BCL2-NLK axis	Maintains proliferation and cytotoxicity; downregulation with aging leads to functional decline	[101]

## 4. Translational Potential: From Biology to Therapy

The intricate regulatory networks woven by ncRNAs across the three core axes—extracellular ncRNAs from the TME regulating NK cells, NK cell-derived EVs modulating TME components, and endogenous ncRNAs regulating intrinsic NK cell fitness—represent potential targets for cancer diagnosis, prognosis, and treatment. The transition from mechanistic understanding to therapeutic application is progressing, propelled by advances in RNA biology and drug delivery platforms. This section critically assesses the translational potential of targeting these ncRNAs to mitigate immunosuppression and bolster NK cell-based immunotherapy.

### 4.1. ncRNAs as Diagnostic and Prognostic Biomarkers

While a vast array of ncRNAs have been mechanistically linked to NK cell dysfunction within the TME, it is crucial to explicitly distinguish between findings strictly confined to in vitro and preclinical in vivo models versus observations validated in human patient cohorts. A substantial portion of the characterized ncRNA regulatory networks—such as the in vitro effects of FENDRR on impairing NK cell recruitment [35,36], the direct suppression of cytotoxicity by secreted miR-23a [43], and the endogenous regulation of homing by miR-126 [86]—currently remain in the preclinical stage, relying primarily on cell lines and murine models. Conversely, a select subset of ncRNAs has demonstrated robust prognostic and clinical relevance through validation in patient cohorts and clinical samples. For example, transcriptomic analyses of clinical bladder cancer cohorts have correlated the m6A-related lncRNA LINC02604 with elevated PD-L1 expression and altered NK cell activation states in patients [40]. Furthermore, in clinical liquid biopsies, elevated circulating levels of MALAT1 reliably predict an immunosuppressive TME and serve as a predictive biomarker for resistance to immune checkpoint inhibitors in patient cohorts [106,107]. Similarly, elevated serum concentrations of the NK cell-derived lncRNA NKILA have been clinically validated to correlate inversely with NK cell functional fitness and signify poor prognosis across multiple malignancies [25]. Bridging this translational gap—from purely mechanistic in vitro observations to rigorous validation in large-scale patient cohorts—is an essential prerequisite for advancing these ncRNAs from bench-side concepts to precision oncology targets.

The relative stability of specific ncRNAs in bodily fluids, their tissue-specific expression patterns, and their dynamic reflection of tumor burden and TME status make them promising candidates for non-invasive liquid biopsies [108]. Differential expression signatures of lncRNAs (e.g., FENDRR, MALAT1, PWAR6) and circRNAs (e.g., CDR1as, hsa_circ_0008305) in plasma or serum have been robustly correlated with cancer type, stage, metastatic risk, and response to therapy. For instance, high levels of EV-derivedMALAT1 in the circulation may indicate an immunosuppressive TME and resistance to immune checkpoint inhibitors, serving as a valuable predictive biomarker [106,107]. Furthermore, the detection of CAF-derived ncRNAs, such as exosomal PWAR6, in patient blood could provide a direct readout of stromal-mediated immunosuppression [18]. Elevated levels of NK cell-derived NKILA in serum correlate with reduced NK cell function and poor prognosis in multiple cancers [25]. The integration of multi-ncRNA panels into diagnostic algorithms holds the potential to move beyond static histopathological classifications towards a dynamic, molecular-level assessment of the TME, guiding personalized therapeutic decisions.

### 4.2. ncRNA-Targeted Therapeutic Strategies

#### 4.2.1. Targeted Intervention Modalities

The direct targeting of oncogenic ncRNAs or the restoration of tumor-suppressive ncRNAs forms the cornerstone of this therapeutic approach. However, to transition these strategies from preclinical observations to viable clinical interventions, each modality must be systematically assessed across a structured evidence-based framework encompassing delivery specificity, biodistribution, pharmacokinetics, immune toxicity, manufacturing challenges, and measurable pharmacodynamic biomarkers of NK cell activation.

Antisense Oligonucleotides (ASOs) and Locked Nucleic Acids (LNAs): These single-stranded DNA analogs bind complementarily to target lncRNAs or circRNAs, triggering their degradation by RNase H or sterically blocking their functional interactions [109,110]. LNAs, with chemical modifications conferring high binding affinity and metabolic stability, can target oncogenic ncRNAs such as FENDRR (tumor-derived), PWAR6 (CAF-derived), and NKILA (NK cell-derived) [111]. ASOs targeting MALAT1 (endothelial cell-derived) have shown promise in preclinical models by enhancing NK cell infiltration [112]. From a clinical feasibility perspective, a major advantage of ASOs and LNAs is their amenability to scalable, solid-phase chemical synthesis, making GMP manufacturing relatively straightforward and cost-effective [113]. Furthermore, structural modifications (e.g., 2′-O-methylation, phosphorothioate backbones) significantly extend their circulating pharmacokinetics from minutes to days or weeks, while effectively mitigating the immune toxicity (such as unintended Toll-like receptor activation) often seen with unmodified oligonucleotides [114]. However, systemic administration of unconjugated ASOs and LNAs still suffers from poor delivery specificity; they predominantly accumulate in the reticuloendothelial system, particularly the liver and kidneys, thereby restricting effective biodistribution and therapeutic concentrations within the extrahepatic tumor microenvironment [115].

Small Interfering RNAs (siRNAs): siRNAs can be designed to specifically cleave and degrade complementary oncogenic ncRNA transcripts [116]. SiRNAs targeting CCAT1 (CAF-derived) and CDR1as (macrophage-derived) have been shown to restore NK cell function in preclinical models [47,117]. The major challenge remains the efficient and targeted delivery of these molecules to the relevant cell types within the TME [118]. They lack inherent tissue specificity, fail to penetrate cellular membranes efficiently, and are rapidly eliminated via renal clearance [119]. Consequently, their unformulated plasma half-life is typically less than an hour due to ubiquitous ribonucleases [119]. Moreover, as potent pathogen-associated molecular patterns (PAMPs), unshielded double-stranded siRNAs risk severe immune toxicity by triggering endosomal TLR3/7/8-mediated systemic cytokine storms [120]. Therefore, while synthesizing the bare siRNA sequence is simple, advancing them to the clinic strictly requires formulation into stable, uniform clinical-grade delivery vehicles, which significantly escalates the manufacturing complexity [121].

Nanoparticle-Based Delivery Systems: For tumor-suppressive ncRNAs lost in cancer, therapeutic restoration can be achieved by delivering synthetic mimics or using gene therapy vectors. MiR-155 mimics, delivered via NK cell-targeted nanoparticles, enhance NK cell activation and cytotoxicity [68,122]. Viral vectors expressing CARMN or GAS5 have been used to restore NK cell function in preclinical models [63,88]. Encapsulation not only drastically extends the circulation time but also facilitates crucial endosomal escape within target cells [123]. While LNP production benefits from highly scalable microfluidic mixing techniques, standard LNPs inherently skew toward hepatic accumulation via apolipoprotein binding. Achieving precise TME or NK-cell delivery specificity demands active surface functionalization (e.g., with NKG2D-targeting peptides or α-SMA antibodies) [18,124]. Furthermore, synthetic LNPs carry a potential risk of complement activation-related pseudoallergy (CARPA) or hepatotoxicity. In contrast, engineered autologous EVs exhibit superior biocompatibility and immune privilege, minimizing off-target immunotoxicity [124]. However, the clinical translation of EV-based systems is currently bottlenecked by formidable manufacturing challenges, primarily low extraction yields, high batch-to-batch heterogeneity, and complex purification requirements [124].

Measurable Pharmacodynamic Biomarkers of NK Cell Activation: Ultimately, validating the clinical efficacy of any of these ncRNA-targeted interventions requires establishing measurable pharmacodynamic biomarkers to confirm target engagement and functional NK cell reinvigoration. Rather than relying solely on lagging indicators like macroscopic tumor shrinkage, early immune activation should be dynamically monitored using liquid biopsies or tumor-infiltrating lymphocyte (TIL) profiling. The translational feasibility of these therapies relies heavily on quantifying the upregulation of surface activating receptors (e.g., NKG2D, DNAM-1, NKp46) and degranulation markers (CD107a) on circulating and intratumoral NK cell populations. Additionally, the functional reversal of TME-induced exhaustion must be validated by measuring augmented local or systemic secretion of effector molecules, specifically IFN-γ, perforin, and granzyme B [125,126].

#### 4.2.2. Synergistic Combination Therapies

Monotherapy with ncRNA-targeting agents may face limitations due to the robustness of oncogenic networks [127]. Therefore, combining them with established modalities presents a highly promising avenue.

With Immune Checkpoint Inhibitors (ICIs): Targeting an immunosuppressive ncRNA like HOTAIR could potentially upregulate NKG2D ligands on tumor cells, making them recognizable to NK cells, while simultaneously, anti-PD-1/PD-L1 antibodies reinvigorate exhausted T cells [128]. This dual attack on different immune evasion mechanisms can lead to synergistic antitumor efficacy.

With Chemotherapy/Radiotherapy: Certain chemotherapeutic agents and radiation can induce the expression of stress ligands on tumor cells, making them better targets for NK cells [93,94]. Silencing an ncRNA that dampens this stress response could thereby enhance the efficacy of these conventional treatments [94].

With Cell-Based Therapies: Engineering NK or CAR-NK cells to express siRNAs or ASOs targeting key immunosuppressive ncRNAs (e.g., NKILA) within their immediate microenvironment could create “armored” immune cells that are resistant to TME-derived suppression [129,130,131], thereby improving their persistence and cytotoxic activity [130].

#### 4.2.3. Distinct Biochemical and Structural Modalities of ncRNA Classes as Therapeutic Targets

To effectively translate ncRNA-based discoveries into viable clinical interventions within the TME, a nuanced understanding of the intrinsic biochemical properties and structural topologies governing different ncRNA classes is imperative. Distinct categories of ncRNAs—namely miRNAs, lncRNAs, and circRNAs—exhibit profound differences in their stability, target specificity, and susceptibility to delivery vehicles, which ultimately dictate their therapeutic feasibility [132]. For instance, while miRNAs are structurally amenable to synthetic mimics or antagomirs, their propensity for seed-sequence-dependent binding introduces a substantial risk of off-target transcriptomic disruptions [133]. Conversely, lncRNAs offer high sequence specificity but pose formidable challenges due to their large macromolecular size and intricate secondary/tertiary folding, which often impede efficient intracellular delivery and oligonucleotide binding [134]. Emerging as unique therapeutic candidates, circRNAs benefit from a covalently closed loop structure that inherently confers resistance to exonucleolytic degradation, resulting in a prolonged biological half-life [135]. However, therapeutic modulation of circRNAs remains technically constrained by the complexity of delivering large expression vectors or synthesizing stable artificial circular constructs without eliciting innate immune responses. A systematic comparative analysis of these parameters is delineated below to guide rational drug design for NK cell-directed therapies.

### 4.3. Preclinical and Clinical Progress and Challenges

The translational pipeline is beginning to yield tangible outcomes. To date, several ncRNA-targeted therapeutics have advanced into clinical development for cancer and viral indications; however, it is critical to clarify that their primary clinical mechanisms of action are general cancer therapies, and their specific relevance to NK cell–TME crosstalk remains secondary or strictly preclinical. BC-819 (DTA-H19), a DNA plasmid leveraging the lncRNA H19 gene promoter to drive diphtheria toxin A expression in H19-positive tumor cells [55]. While evaluated in phase I/II clinical trials for recurrent bladder and ovarian cancers with manageable safety profiles [136,137], this agent relies on general tumor-specific transcription for direct cytotoxicity, rather than actively modulating NK-regulatory ncRNA networks.

MRG-106 (Cobomarsen), a targeted inhibitor of miR-155, has progressed to phase II clinical trials for the treatment of cutaneous T-cell lymphoma (CTCL) and peripheral T-cell lymphoma (PTCL). Clinical data indicate that MRG-106 improves the objective response rate in CTCL patients [138]. Its primary goal is altering tumor cell survival; any capacity to modulate immune cell function or NK cell activation represents a secondary mechanism extrapolated from preclinical TME models.

RG-012, a therapeutic agent targeting lncRNA MALAT1, is currently under phase I/II clinical evaluation for non-small cell lung cancer (NSCLC) and metastatic breast cancer. Preclinical studies have confirmed that RG-012 can enhance NK cell infiltration into the tumor parenchyma and potentiate antitumor immune responses by disrupting the immunosuppressive regulatory network mediated by MALAT1 [139], but its clinical development is broadly aimed at transcriptomic reprogramming, and its direct impact on human NK cell immunity remains to be established.

Miravirsen, a miR-122 inhibitor initially developed for viral hepatitis, has been repurposed for hepatocellular carcinoma (HCC) and completed phase II clinical trials [140]. Assumed mechanistically, Miravirsen regulates the crosstalk between NK cells and tumor cells by targeting miR-122, thereby inhibiting tumor progression [141]. Clinical evaluations have demonstrated a favorable safety profile with no severe treatment-related adverse events, supporting its continued development as a novel immunotherapeutic strategy for HCC.

Despite promising clinical advancements, translating ncRNA therapeutics is bottlenecked by formidable in vivo delivery challenges. Primarily, unmodified RNA molecules suffer from exceptionally short biological half-lives due to ubiquitous circulating RNases and rapid renal clearance [128,139]. Overcoming this instability necessitates extensive chemical modifications (e.g., 2′-O-methylation, phosphorothioate backbones) integrated with protective delivery vehicles. Even if systemic stability is achieved, strict tissue-specific targeting presents a profound physical hurdle [139]. Specifically, the TME is characteristically dense and fibrotic, with elevated interstitial fluid pressure that severely restricts the extravasation and deep parenchymal penetration of therapeutic RNAs. Furthermore, these exogenous transcripts risk inadvertent recognition by endosomal pattern recognition receptors, such as Toll-like receptors (TLRs), which can trigger unintended innate immune cascades leading to systemic cytokine storms or hepatotoxicity [128,139].

To circumvent these physiological barriers, advanced delivery vehicles are actively being optimized, though each presents distinct limitations and opportunities for innovation. Currently, lipid nanoparticles (LNPs) serve as the clinical gold standard for RNA delivery, providing excellent transcript protection [121]. However, standard LNPs inherently accumulate in reticuloendothelial organs—predominantly the liver and spleen—via apolipoprotein E-mediated uptake, thereby limiting their utility for extrahepatic tumors and increasing the risk of off-target accumulation. To overcome this, recent advances focus on conjugating active targeting ligands, such as NKG2D-targeting peptides or anti-fibroblast activation protein antibodies, and optimizing the structural geometry of ionizable lipids to facilitate specific TME accumulation and efficient endosomal escape [123]. Alternatively, naturally occurring exosome (EV)-based delivery systems offer superior biocompatibility, immune privilege, and inherent cellular tropism compared to synthetic nanoparticles [124]. While their clinical translation has historically been hindered by manufacturing limitations, including low extraction yields and batch-to-batch heterogeneity, recent bioengineering breakthroughs have leveraged engineered EVs to bypass these bottlenecks. For instance, decorating EVs with the “don’t eat me” signal CD47 allows them to evade macrophage clearance, whereas anchoring specific single-chain variable fragments (ScFvs) to the exosomal membrane enables highly precise targeting of tumor or stromal cells, thereby minimizing systemic toxicity and maximizing localized NK cell rescue [142]. Addressing these challenges through interdisciplinary collaboration is essential for fully realizing the potential of ncRNA-targeted therapies to reprogram the TME and unlock the power of NK cell immunity.

Beyond delivery hurdles, the profound impact of tumor heterogeneity poses a major challenge to the clinical efficacy of ncRNA-targeted therapeutic strategies [143]. Inter-patient heterogeneity dictates that the dominant immunosuppressive ncRNA networks vary significantly among individuals, even within the same cancer histological subtype [144]. For instance, while NK cell dysfunction in one patient may be driven primarily by CAF-derived PWAR6, another patient’s tumor may rely predominantly on tumor-derived SNHG10 to impair NK cell cytotoxicity. This inter-patient variability renders “one-size-fits-all” approaches ineffective, necessitating rigorous personalized transcriptomic profiling (such as liquid biopsies of EV-derived ncRNAs) to identify the specific actionable ncRNA nodes prior to intervention. Furthermore, intra-tumoral heterogeneity—the spatial and temporal diversity of cell sub-clones within a single tumor—creates distinct immunosuppressive micro-niches [145]. Hypoxic core regions may upregulate specific pathways (e.g., miR-210 induction) to restrict NK cell survival, whereas the invasive margins might utilize divergent exosomal ncRNA signals. Consequently, therapeutically silencing a single ncRNA target may only rescue NK cell function in restricted spatial niches. Sub-clones in adjacent regions not dependent on the targeted ncRNA will evade immunosurveillance, survive, and inevitably drive relapse [144]. Overcoming this spatial heterogeneity requires multiplexed targeting strategies—simultaneously modulating multiple ncRNA nodes—or combining ncRNA interventions with broad-spectrum immune checkpoint inhibitors to counteract regional immune evasion globally.

Furthermore, the emergence of resistance to ncRNA-targeted therapies represents a critical, yet underexplored, clinical challenge. Tumors possess highly plastic transcriptomic landscapes; thus, therapeutically silencing a single oncogenic ncRNA may rapidly induce compensatory mechanisms. For instance, inhibiting a specific miRNA could trigger the compensatory activation of alternative ceRNA networks or upregulate redundant signaling pathways that bypass the therapeutic blockade [146]. Additionally, tumor cells may develop resistance through genomic mutations in specific ncRNA binding sites [147,148] or through profound alterations in the endogenous RNA processing machinery (e.g., Dicer, Drosha, or specific RNA-binding proteins) [149], ultimately restoring the immunosuppressive integrity of the TME [150,151].

## 5. Conclusions and Future Perspectives

### 5.1. Systems-Level Synthesis: Network Architecture and Core Regulatory Hubs

The intricate intercellular dialogue between NK cells and the TME transcends the linear regulation of isolated genes; it operates as a highly dynamic, spatially organized, and multidimensional ncRNA network. A systems-level synthesis of current evidence reveals that diverse transcriptomic elements—whether packaged in EVs or acting intrinsically—ultimately converge upon a defined set of core regulatory hubs and convergent signaling pathways to dictate immune evasion.

Convergent Signaling Hubs: Regardless of their cellular origin (e.g., tumor cells, cancer-associated fibroblasts, or exhausted NK cells), extracellular and endogenous ncRNAs consistently hijack specific signaling bottlenecks. The NF-κB and JAK-STAT pathways emerge as universal master integrators within this network [33,49,58,68]. Oncogenic ncRNAs systematically exploit these hubs to suppress IFN-γ production and impair cytotoxic degranulation, while simultaneously amplifying the secretion of immunosuppressive cytokines.

Metabolic Checkpoints as Structural Nodes: Metabolic reprogramming is a fundamental architect of ncRNA networks. Key regulatory axes heavily target metabolic checkpoints, notably the PTEN/PI3K/AKT cascade and HIF-1α-driven adaptation programs [39,52,62,96]. By manipulating these nodes, ncRNAs rigidly control the transition of NK cells between oxidative phosphorylation and glycolysis, functioning as a “metabolic switch” that drives cells toward terminal exhaustion under chronic TME stress.

Bifurcated Network Architecture: The regulatory architecture is defined by a spatiotemporal bifurcation. In the cytoplasm, ceRNA networks act as rapid, post-transcriptional “buffers” regulating receptor expression and immediate effector functions. Conversely, in the nucleus, lncRNAs orchestrate profound, long-term phenotypic locking via epigenetic remodeling hubs (e.g., the PRC2 and EZH2 complexes) [29,30,31,104,105]. This structural duality enables the TME to induce both acute NK cell suppression and irreversible functional exhaustion.

### 5.2. Core Unresolved Biological Questions

Despite the considerable progress, several challenges and exciting frontiers lie ahead, charting the course for future research.

Despite the explosive cataloging of novel ncRNAs, translating these discoveries into robust clinical applications is fundamentally bottlenecked by several unresolved biological questions that must define the field’s future trajectory.

The Stoichiometric Reality of ceRNA Networks In Vivo: A massive translational chasm exists regarding the physiological authenticity of ceRNA crosstalk. The absolute intracellular abundance of most endogenous lncRNAs and circRNAs is often dwarfed by their targeted miRNAs [26,27,28,29]. It remains a critical, unresolved question whether the EV-mediated transfer of ncRNAs can truly achieve the stoichiometric thresholds required for functional target occupancy within the diffusion-limited architecture of the human TME, or if many current observations are largely artifacts of supraphysiological in vitro overexpression models.

Mechanisms of State-Dependent EV Cargo Sorting: The secretory profile of NK cells is highly pleiotropic, actively shifting from tumor-suppressive to pro-tumorigenic based on their metabolic fitness [80,81]. The precise intracellular sensors and RNA-binding proteins that physically rewire this selective EV sorting machinery under hypoxic and acidic TME stress remain a profound “black box” in tumor immunology.

Spatiotemporal Plasticity and Subcellular Shuttling: The field lacks a mechanistic understanding of how environmental cues trigger the subcellular relocalization of endogenous ncRNAs [104,105]. How a single transcript structurally transforms from a cytoplasmic survival factor into a nuclear epigenetic exhaustion driver in response to chronic antigen stimulation is largely unmapped.

Transcriptomic Plasticity and Acquired Resistance: Tumor intra-tumoral heterogeneity poses a severe threat to ncRNA-targeted monotherapies. We have yet to fully decode the compensatory transcriptomic networks, alternative ceRNA circuits, or genomic mutations [144,145,146,147] that tumors inevitably activate when a dominant oncogenic ncRNA node is therapeutically silenced.

### 5.3. Future Perspectives: Towards Precision Immunotherapy

Overcoming these biological hurdles demands a paradigm shift from simple molecular cataloging to high-resolution, multiplexed spatial mapping. Future breakthroughs rely heavily on the integration of single-cell RNA sequencing (scRNA-seq) with spatial transcriptomics [152,153,154]. This multi-omics approach is mandatory to dismantle the averaging artifacts of bulk RNA-seq, allowing researchers to mathematically predict dominant, micro-niche-specific ceRNA networks and resolve the exact cellular origins of immunosuppressive secretomes at the invasive margin.

Concurrently, the clinical future of ncRNA modulators hinges on overcoming formidable in vivo delivery barriers [155]. The evolution of delivery platforms—transitioning from non-specific LNPs to bioengineered, tissue-responsive EVs conjugated with active targeting ligands [123,124,142]—will dictate the feasibility of strict tissue-specific transcriptomic reprogramming. Ultimately, by modulating these core ncRNA regulatory hubs in synergy with immune checkpoint blockade, we can transition from reactive interventions to the proactive reprogramming of the TME, thereby unlocking the full, durable potential of NK cell-directed immunotherapies.

## Data Availability

No new data were created or analyzed in this study. Data sharing is not applicable to this article.

## Abbreviations

The following abbreviations are used in this manuscript:

3′-UTR	3′-untranslated region
ASOs	Antisense Oligonucleotides
CAFs	Cancer-associated fibroblasts
ceRNA	Competitive endogenous RNA
circRNAs	Circular RNAs
CTCL	Cutaneous T-cell lymphoma
DCs	Dendritic cells
ECM	Extracellular matrix
EMT	Epithelial–mesenchymal transition
EVs	Extracellular vesicles
H3K27me3	Histone H3 lysine 27 trimethylation
HCC	Hepatocellular carcinoma
ICIs	Immune Checkpoint Inhibitors
LNAs	Locked Nucleic Acids
LNPs	Lipid nanoparticles
lncRNAs	Long non-coding RNAs
MDSCs	Myeloid-derived suppressor cells
miRNAs	MicroRNAs
MREs	MicroRNA response elements
ncRNAs	Non-coding RNAs
NK	Natural killer
NSCLC	Non-small cell lung cancer
PAMPs	Pathogen-associated molecular patterns
PRC2	Polycomb Repressive Complex 2
PTCL	Peripheral T-cell lymphoma
qCAF	Quiescent cancer-associated fibroblast
scRNA-seq	Single-cell RNA sequencing
ScFvs	Single-chain variable fragments
siRNAs	Small interfering RNAs
TAMs	Tumor-associated macrophages
TLRs	Toll-like receptors
TME	Tumor microenvironment
Tregs	Regulatory T cells
